# Interoccasion variability in population pharmacokinetic models: identifiability, influence, interdependencies and derived study design recommendations

**DOI:** 10.1007/s10928-025-09966-7

**Published:** 2025-04-11

**Authors:** Emily Behrens, Sebastian G. Wicha

**Affiliations:** Department of Clinical Pharmacy, Institute of Pharmacy, University of Hamburg, Bundesstraße 45, 20146 Hamburg, Germany

**Keywords:** Interoccasion variability, Study design, Pharmacometrics, Stochastic simulation and estimation, NONMEM^®^

## Abstract

Modeling interoccasion variability (IOV) of pharmacokinetic parameters is challenging in sparse study designs. We conducted a simulation study with stochastic simulation and estimation (SSE) to evaluate the influence of IOV (25, 75%CV) from numerous perspectives (power, type I error, accuracy and precision of parameter estimates, consequences of neglecting an IOV, capability to detect the ‘correct’ IOV). To expand the scope from modeling-related aspects to clinical trial practice, we investigated the minimal sample size for IOV detection and calculated areas under the concentration-time curve (AUC) derived from models containing IOV and mis-specified models. The power to correctly detect an IOV increased from one to three occasions (OCC) and the type I error rate to falsely include an IOV was not elevated. Two sampling schemes were compared (with/without trough sample) and including a trough sample resulted in better performance throughout the different evaluations in this simulation study. Parameters were estimated more precisely when more OCCs were included and IOV was of high effect size. Neglecting an IOV that was truly present had a high impact on bias and imprecision of the parameter estimates, mostly on interindividual variabilities and residual error. To reach a power of ≥ 95% in all scenarios when sampling in three OCCs between 10 and 50 patients were required in the investigated setting. AUC calculations with mis-specified models revealed a distorted AUC distribution as IOV was not considered.

**Supplementary Information:**

The online version contains supplementary material available at 10.1007/s10928-025-09966-7.

## Introduction

Nonlinear mixed-effect modeling aims at identifying and explaining different types of random effects within the model. In principle, two levels of random effects can be distinguished: Karlsson and Sheiner referred to the first level of random effects as the “random variation in parameters” and described the second level as “random variation of observations” [1]. Parameter variability is further subdivided into interindividual variability (IIV) and an intraindividual variability between different occasions (OCC), interoccasion variability (IOV) [1]. The importance of the evaluation and inclusion (if applicable) of IOV has been emphasized repeatedly from a pharmacokinetic (PK) but also pharmacodynamic (PD) perspective [1–3].

Sampling schemes to inform PK modeling in e.g. phase II clinical studies are sparse [4, 5]. Therefore, sampling may only be performed in a single dosing interval. Conceptionally, sampling in one OCC is not sufficient for the quantification of IOV as it represents the variability between different OCCs. However, IOV is often intrinsically present and influential at the time of the first observed OCC, even though in model development pharmacometricians often neglect testing for IOV given the sampling scheme constraints mentioned above.

Our simulation study aimed at exploration and evaluation of the influence of IOV in typical sparse sampling settings of phase II clinical studies using stochastic simulation and estimation (SSE). In a first step, we evaluated different sampling scenarios to evaluate power and type I error concerning the capability of finding an IOV in an estimation given that the IOV is truly present on the one hand and the risk of falsely including an IOV in a PK model on the other hand. In a second step, the influence of a truly present IOV on accuracy and precision of parameter estimates was explored. In addition, we investigated if the correct (simulated) IOV would be detectable when models including an IOV on a different parameter were used for estimation during e.g. model building. Moreover, the influence of an intentionally ignored IOV was evaluated. We performed sample size calculations using an SSE workflow with increasing number of patients represented in the datasets to evaluate the relationship between sample size and power outcome. To demonstrate clinical relevance, we calculated areas under the concentration-time curve (AUC) of mis-specified models and compared them to AUCs from correctly informed models.

## Methods

**Fig. 1:**
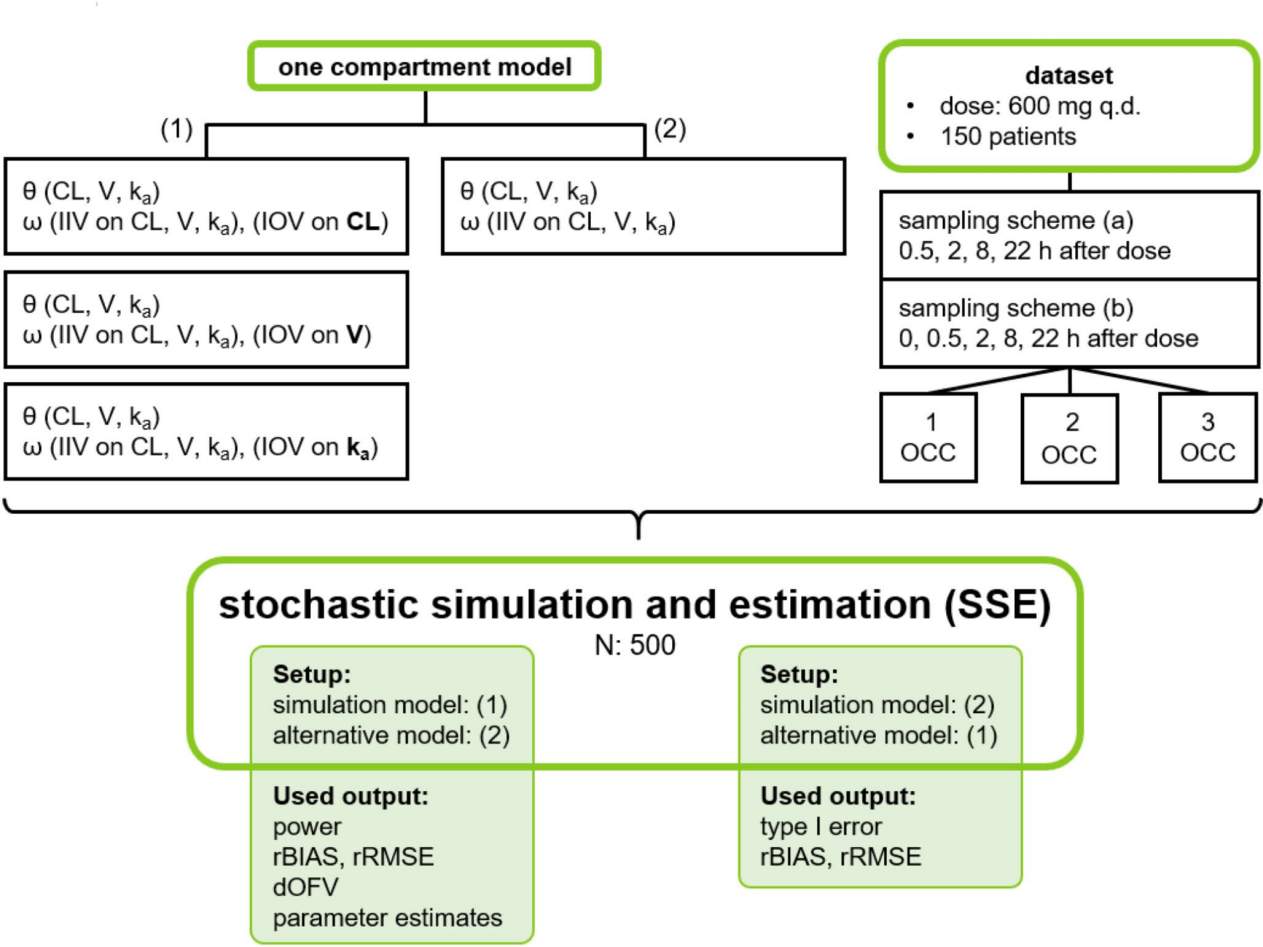
Graphical workflow of the simulation study with evaluated scenarios and sampling schemes (θ: fixed effects, ω: random effects, CL: clearance, IIV: interindividual variability, IOV: interoccasion variability, k_a_: absorption constant, OCC: occasion, rBIAS: relative bias, rRMSE: relative root mean squared error, V: volume of distribution)

A graphical workflow of the simulation study is shown in Fig. 1. To test and evaluate different hypotheses, SSEs were executed and power and type I error calculations were performed while assessing parameter bias and imprecision simultaneously. As the focus was set on the evaluation of the influence of IOV, datasets were generated that feature PK sampling in one to three dosing OCCs. The following section is a detailed description of each component or function of the simulation study.

### Model

The simulation study was conducted using and adapting the one compartment model describing the population PK of linezolid in multidrug-resistant tuberculosis patients previously published by Tietjen et al. [6]. The interindividual variabilities on clearance (CL), volume of distribution (V) and absorption constant (k_a_) were set to 32%CV (named IIV_CL/V/ka_ in the following). The model solely containing IIVs is referred to as IIV_only_. Implementing an IOV on one parameter of the IIV_only_ model at a time led to three different models referenced as IOV_CL_, IOV_V_ and IOV_ka_ in the following. Two magnitudes of IOV were implemented in each case, 25%CV (IOV25) and 75%CV (IOV75). For the sake of traceability, e.g. IOV25 on CL will be described as IOV25_CL_. In total, seven various models were created (IOV25_CL_, IOV75_CL_, IOV25_V_, IOV75_V_, IOV25_ka_, IOV75_ka_, IIV_only_). The implementation of the IOV was encoded in compliance with the approach described by Karlsson and Sheiner [1]. ETAs were assigned to every occasion in the dataset. The residual variability was described by a combined proportional and additive error model [6].

The parameter values used in the model file are listed in Table 1.

**Table 1:** Initial parameter estimates of the population pharmacokinetic model used in the simulation study (CL: clearance, CV: coefficient of variation, IIV: interindividual variability, IOV: interoccasion variability, k_a_: absorption rate constant, V: volume of distribution)

Structural parameters	Parameter value
CL [L/h]	6.8
V [L]	38.9
k_a_ [h^− 1^]	0.617
IIV/IOV^a^	
IIV CL [%CV]	32
IIV V [%CV]	32
IIV k_a_ [%CV]	32
IOV_CL_ or IOV_V_ or IOV_ka_ [%CV]	25 and 75
Residual variability	
Proportional error [%]	19.1
Additive error [mg/L]	0.56

^a^Equation used for transformation of ω^2^ to %CV: $\:\%CV\:=\:\sqrt{{e}^{{{\upomega\:}}^{2}\:}-1}\:\times\:100$

### Sampling schemes

The $DESIGN feature in NONMEM^®^ was used to evaluate an optimized study design for the administered daily dose of 600 mg based on the D-optimality criterion [7]. The starting point for the optimization was the IOV25_CL_ model file. The design element to be optimized was time since the first dosing event (TIME) and the dataset that sets the initial timepoints for the optimization process contained five sampling timepoints. The initial sampling times were provided their own stratification variable (TSTRAT) values to assure an independent variation while optimizing TIME. The boundaries for TIME were 72.5 (TMIN) and 96 (TMAX). TMIN was chosen to reflect steady-state. The $DESIGN tool suggested five samples with sampling at 72.5 h twice and three subsequent samples. This led to the design of sampling scheme (a) with a total of four samples. For sampling scheme (b) a sample right before the timepoint of dosing was added to the dataset manually. Hence, sampling scheme (b) includes a trough concentration informed by the previous occasion.

Ultimately, two sampling schemes were evaluated: (a) 0.5, 2, 8, 22 h after dose and (b) 0/pre-dose, 0.5, 2, 8, 22 h after dose.

### Dataset generation

Three simulation datasets were generated using R. One dataset contained samples taken at only one OCC. Another dataset contained samples taken in two dosing OCCs and the last dataset contained samples taken in three dosing OCCs. Dosing events with and without observations were considered an OCC; the added trough sample in sampling scheme (b) belongs to the previous OCC. As OCC is not a predefined variable in NONMEN, it hast to be understood and treated like a time-varying covariate. Analogous to the approach taken by Denti, “dummy records” (EVID = 2) were used to ensure the correct assignment of the OCC number within one dosing interval [8]. A dataset example of one patient for each sampling scheme can be found in the supplementary files (Figure S1).

A daily dose of 600 mg was simulated for 150 patients. The number of patients in phase II studies varies depending on the study design. A total of 150 patients was chosen as a conceivable number of patients from real life phase II studies [9, 10]. Once the dataset framework entered the SSE workflow, it was completed by simulated DVs.

### Stochastic simulation and estimation

**Fig. 2:**
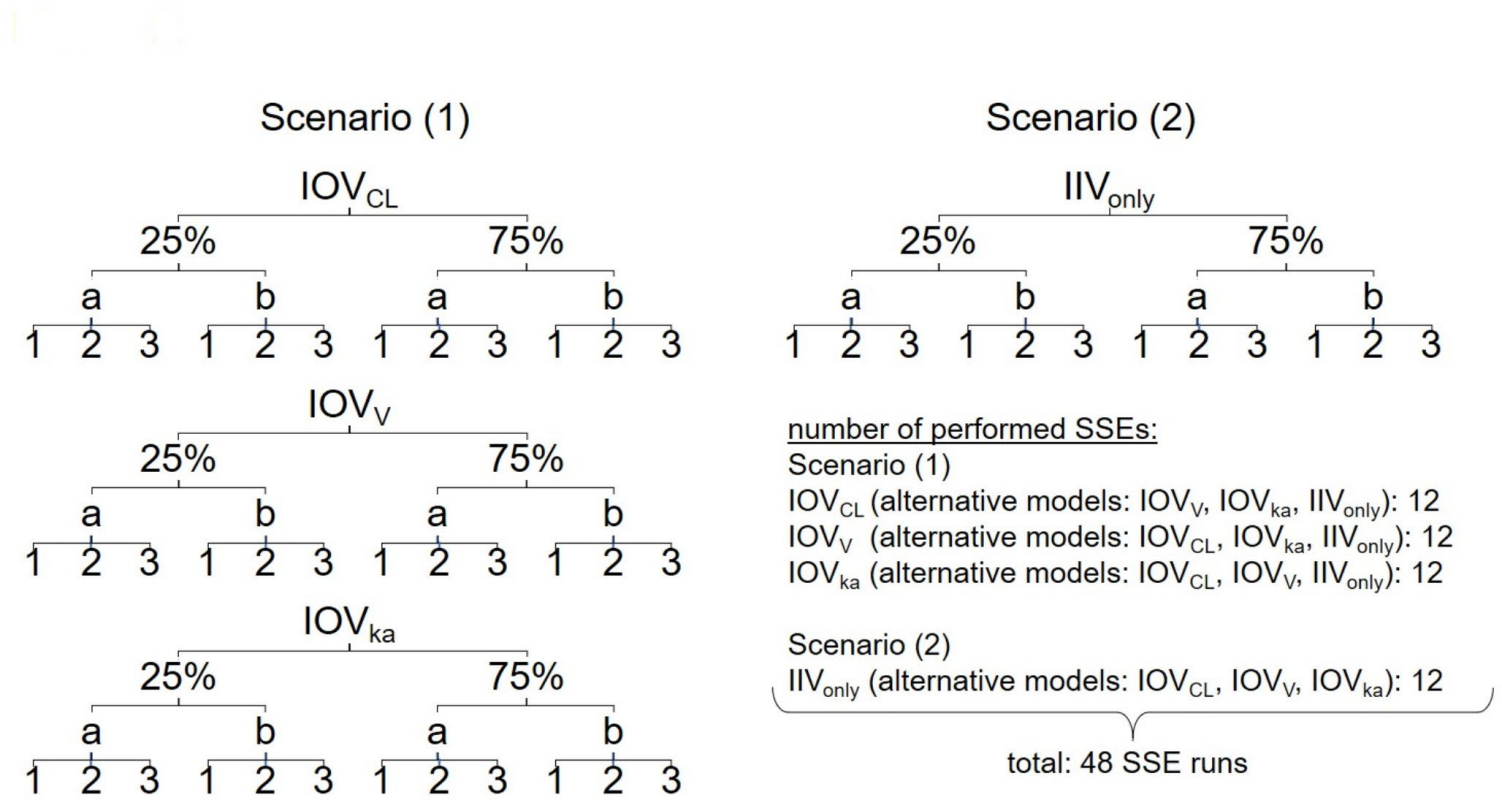
Summary of evaluated SSE runs (a: sampling scheme (a), b: sampling scheme (b), 1: one occasion, 2: two occasions, 3: three occasions, IOV_CL_: interoccasion variability on clearance, IOV_V_: interoccasion variability on volume of distribution, IOV_ka_: interoccasion variability on absorption constant, SSE: stochastic simulation and estimation) with scenario (1) showing the power setup when IIV_only_ was chosen as the alternative model (alternative models containing IOV for calculation of difference in objective function value, explained in Identification of correct IOV), while scenario (2) illustrates the setup for type I error calculations

The SSE tool from Perl-speaks-NONMEM (PsN) was used for the calculation of the power or type I error to find an IOV that truly is or is not present, respectively [11]. The probability of correctly rejecting the null hypothesis is defined as the power of a statistical test, while type I error describes the probability of falsely rejecting the null hypothesis. The different combinations of simulation and estimation models that were used for the power or type I error setup are shown in Fig. 1. The number of simulated datasets to generate in the SSE was set to 500 (N in Fig. 1). Figure 2 gives an overview of the number of different SSE runs that were performed in total except for the SSE runs used for the minimal sample size investigation. We used seven different model files IOV25_CL_, IOV75_CL_, IOV25_V_, IOV75_V_, IOV25_ka_, IOV75_ka_ and IIV_only_. For power calculations 36 SSEs were performed and we calibrated the chi-square critical value to an alpha of 0.05. Table S2 shows the critical values from the alpha-calibration. The SSEs from the power calculations were also used for the assessment of the ability to identify the correct IOV. Another 12 SSEs were performed to calculate type I errors. The total number of SSEs performed was 48 (except for minimal sample size SSEs), while using multiple alternative models in the SSE command. Additionally, 192 SSE runs were executed for the determination of the minimal sample size. The same initial parameter values were used for the simulation and the estimation step.

The SSE tool from Perl-speaks-NONMEM (PsN) was used for the calculation of the power or type I error to find an IOV that truly is or is not present, respectively [11]. The probability of correctly rejecting the null hypothesis is defined as the power of a statistical test, while type I error describes the probability of falsely rejecting the null hypothesis. The different combinations of simulation and estimation models that were used for the power or type I error setup are shown in Fig. 1. The number of simulated datasets to generate in the SSE was set to 500 (N in Fig. 1). Figure 2 gives an overview of the number of different SSE runs that were performed in total except for the SSE runs used for the minimal sample size investigation. We used seven different model files IOV25_CL_, IOV75_CL_, IOV25_V_, IOV75_V_, IOV25_ka_, IOV75_ka_ and IIV_only_. For power calculations 36 SSEs were performed and we calibrated the chi-square critical value to an alpha of 0.05. Table S2 shows the critical values from the alpha-calibration. The SSEs from the power calculations were also used for the assessment of the ability to identify the correct IOV. Another 12 SSEs were performed to calculate type I errors. The total number of SSEs performed was 48 (except for minimal sample size SSEs), while using multiple alternative models in the SSE command. Additionally, 192 SSE runs were executed for the determination of the minimal sample size. The same initial parameter values were used for the simulation and the estimation step.

### Identification of correct IOV

To assess the ability of identifying the correct IOV, we added several alternative models in the estimation step of the SSE. Therefore, we used a model including IOV in every occasion in the simulation step and models including IOV on structural parameters, which were implemented on different parameters as compared to the simulation model, and IIV_only_ in the estimation step. We calculated the difference between the objective function value (ΔOFV) from the estimated IIV_only_ model and the IOV models to mimic the decision-making process of whether to integrate an IOV in a model or not based on ΔOFV. Negative values of ΔOFV are a result of higher objective function values (OFV) of the IOV model compared to the IIV_only_ model. In general, a lower OFV is an indicator of better model fit and the ΔOFV is considered statistically significant when ΔOFV ≥ critical value. The critical values after alpha-calibration (α = 0.05) can be found in the supplementary files (Table S2).

### Impact of neglecting IOV

We also investigated the impact of a simulated IOV that is not represented in the estimation model of an SSE on the accuracy and imprecision of model parameters. For this purpose, we set up two different scenarios: The first scenario (IOV_included_) consisted of one model used for both the simulation and estimation step. The second scenario (IOV_excluded_) used a simulation model including an IOV and an estimation model that did not include an IOV. Therefore, we could simulate and analyze the impact of ignoring an IOV that is truly present in the simulated data. The SSE runs needed for this evaluation are shown under Scenario (1) in Fig. 2 (e.g. IOV_CL, included_ translates to IOV_CL_ as simulation/estimation model, IOV_CL, excluded_ translates to IOV_CL_ as simulation model and IIV_only_ as estimation/alternative model).

### Minimal sample size investigation

We investigated the relationship between power and the number of patients in the simulation study. The ‘full model’ was a model with an implemented IOV and as the ‘reduced model’ we used the IIV_only_ model, similarly as described above (power setup). The same dataset structure as in the SSE workflow was used, starting with 10 patients up to a total 150 in steps of 10. Sample sizes reaching 95% power were considered sufficient.

### Application example: AUC distribution

After evaluating the effect of neglecting IOV regarding parameter estimation and variability distribution, we further investigated the consequences of applying mis-specified models to demonstrate the potential clinical relevance. Hence, we calculated the AUC (Eq. 1) after simulating with different IOV_CL_ models in one to three OCCs.

1 $$\:AUC=\frac{Dose}{Clearance}$$

In total we covered three scenarios:

I. True model containing IOV_CL_: We used the IOV_CL_ model for simulation.
II. True model with final estimates from SSE: We used the re-estimated IOV_CL_ model for simulation.
III. Mis-specified model neglecting IOV_CL_: We used the re-estimated IIV_only_ model for simulation.

The AUC calculations focused on the influence of IOV, residual variability was not considered but was part of the model files in the same way as shown in Table 1.

### Evaluation

For parameter estimation five candidate models were used: the same model as used for simulation, IIV_only_ and IOV25_CL/V/ka_ or IOV75_CL/V/ka_ models with an IOV on another PK parameter than the one used in the simulation model. When a model with IOV was used in the simulation step and for the estimation part of the SSE a model without this particular IOV (IIV_only_) was used, the setup facilitates power calculations (scenario (1), Fig. 2). Thereby, an evaluation of the ability to detect an IOV that is truly present is possible. To evaluate the type I error rate, the simulation was performed with the IIV_only_ model (scenario (2), Fig. 2). In the estimation step models including IOVs were utilized. Moreover, ΔOFVs from estimations of IIV_only_ compared to estimations of IOV_CL_/IOV_V_/IOV_ka_ were analyzed regarding the ability to correctly detect a simulated (‘true’) IOV in a given scenario.

Imprecision and bias, expressed as relative root mean squared error (rRMSE) (Eq. 2) and relative bias (rBIAS) (Eq. 3) of the population parameters, were used as evaluation criteria.

2 $$\:rRMSE=\sqrt{\frac{1}{N}{\sum\limits_{i}}\frac{{({est}_{i}-{true}_{i})}^{2}}{{true}_{i}^{2}}}\times\:100\%$$

3 $$\:rBIAS=\frac{1}{N}{\sum\limits_{i}}\frac{{{est}_{i}-{true}_{i}}}{{true}_{i}}\times\:100\%$$

Power, type I error, rBIAS, rRMSE and ΔOFV values were taken from the respective SSE output files. Overall, we focused on a significance level of α = 0.05. The chi-square critical value was calibrated to correspond to an α of 0.05 for all the scenarios that resulted in a power value < 100% (Table S2). Hence, the ΔOFV that was considered a statistically significant change in OFV varied throughout the different scenarios.

### Software

All data was simulated and analyzed using NONMEM^®^ (version 7.5.0), operated using the PsN (version 5.3.1/5.4.0 (power curves)) SSE [11]. Creation of the different datasets, implementation of the tested sampling schemes and graphical analysis were performed using R (version 4.2.1) and RStudio (version 2022.07.0) [12].

## Results

### Power

The power to correctly detect an IOV increased with a second or third OCC (Table 2). A notable example of this improvement is the power increase from one (18.2%) to two OCCs (100.0%) up to three OCCs (100.0%) for the simulation model including IOV25_ka_ in the evaluation of sampling scheme (b). A single OCC did not facilitate estimation of IOV25 (power ≤ 65.4%).

Moreover, the power to correctly detect an IOV was higher for the scenarios with IOV75 than for IOV25 analogues. For the IOV_CL_ scenario the power increased from 48.8% (IOV25) to 100.0% (IOV75) in one OCC in sampling scheme (a).

Power was overall highest to detect IOV_V_ while an IOV_ka_ showed the lowest values. IOV_CL_, regardless of the extend, was correctly determinable in sampling scheme (b) according to power calculations (100.0%). Sampling scheme (a) resulted in a lower power value in one observed OCC (IOV25, 48.8%).

Overall, sampling scheme (b) which included the trough sample performed better than (a). The advantages of sampling scheme (b) became obvious while inspecting the power values of the first OCC for both sampling schemes. For instance, the power to correctly detect IOV25_CL_ was 48.8% for sampling scheme (a) and 100.0% for sampling scheme (b).

**Table 2:** Power (%) of detecting an IOV when truly present for the two sampling schemes (a: 0.5, 2, 8, 22 h after dose, b: 0, 0.5, 2, 8, 22 h after dose) and two magnitudes of IOV (25%, 75%) on parameters CL, V and k_a_

Sampling	OCC	SIM/EST with IOV_CL_		SIM/EST with IOV_V_		SIM/EST with IOV_ka_	
		25%	75%	25%	75%	25%	75%
		EST with IIV_only_					
		power [%]	power [%]	power [%]	power [%]	power [%]	power [%]
a	1	48.8	100.0	65.4	99.8	19.2	71.4
a	2	100.0	100.0	100.0	100.0	100.0	100.0
a	3	100.0	100.0	100.0	100.0	100.0	100.0
b	1	100.0	100.0	91.6	100.0	18.2	91.8
b	2	100.0	100.0	100.0	100.0	100.0	100.0
b	3	100.0	100.0	100.0	100.0	100.0	100.0

### Type I error

The results of the type I error calculations are shown in Table 3. In contrast to the results from the power calculations, the type I error rates showed no recordable trend. Notably, the type I errors were less than 5% except for IOV75_CL_, sampling scheme (b) in one OCC.

**Table 3:** Type I error (%) of falsely including a variability parameter that was not simulated for the two sampling schemes (a: 0.5, 2, 8, 22 h after dose, b: 0, 0.5, 2, 8, 22 h after dose) and two magnitudes of IOV (25%, 75%) on parameters CL, V and k_a_

Sampling	OCC	SIM/EST with IIV_only_					
		EST with IOV_CL_		EST with IOV_V_		EST with IOV_ka_	
		25%	75%	25%	75%	25%	75%
		type I error [%]	type I error [%]	type I error [%]	type I error [%]	type I error [%]	type I error [%]
a	1	1.4	0.4	1.6	1.8	0.2	0.0
a	2	0.8	0.8	1.8	0.8	1.6	1.0
a	3	0.4	0.4	2.0	1.4	1.0	3.0
b	1	4.6	5.2	0.8	1.8	0.0	0.0
b	2	0.4	1.4	0.6	0.8	2.0	0.8
b	3	1.4	2.0	0.6	1.4	1.0	0.8

### rBIAS

Generally, the rBIAS values (Table 4) showed a trend to underestimate IOV. The absolute values of the rBIAS were highest in case of the single observed OCC (e.g. 33.7% for IOV75_ka_, sampling scheme (a)). For a power outcome of > 80% the highest rBIAS value of the implemented IOV was 6.4% (IOV25_CL_, sampling scheme (b), one OCC) and the lowest − 10.5% (IOV25_V_, sampling scheme (a), one OCC), respectively.

**Table 4:** rBIAS values of IOV when truly present for the two sampling schemes (a: 0.5, 2, 8, 22 h after dose, b: 0, 0.5, 2, 8, 22 h after dose) and two magnitudes of IOV (25%, 75%) on parameters CL, V and k_a_

Sampling	OCC	SIM with IOV_CL_		SIM with IOV_V_		SIM with IOV_ka_	
		25%	75%	25%	75%	25%	75%
		EST with IOV_CL_		EST with IOV_V_		EST with IOV_ka_	
		rBIAS [%]	rBIAS [%]	rBIAS [%]	rBIAS [%]	rBIAS [%]	rBIAS [%]
a	1	−14.2	3.5	−12.5	−9.7	−20.0	−33.7
a	2	−1.1	−0.0	−2.0	−6.7	1.1	−3.0
a	3	−0.6	−8.0	−2.8	−6.9	−0.0	−2.6
b	1	6.4	−7.7	−10.5	−9.9	7.9	−5.3
b	2	−2.7	−8.6	−3.1	−9.3	1.5	0.0
b	3	−2.2	−8.6	−3.8	−9.6	−0.0	0.0

### rRMSE

The rRMSE values of the estimated IOV decreased with an increasing number of observed OCCs (Table 5, e.g. IOV25_CL_, sampling scheme (a): 72.2% (one OCC), 17.9% (two OCCs), 12.7% (three OCCs)). Overall, comparing IOV25 and IOV75, IOV25 reached higher rRMSE values. For instance, IOV25_CL_ in one OCC led to an rRMSE value of 72.2% in sampling scheme (a) and for the same setup with IOV75_CL_ to an rRMSE value of 24.2%. IOV_CL_ and IOV_V_ resulted in similar rRMSE values, while IOV_ka_ yielded higher rRMSE values (e.g. sampling scheme (a), one OCC, IOV25_CL_ = 62.6%, IOV25_V_ = 58.8%, IOV25_ka_ = 116.7%).

Comparing the two sampling schemes it becomes apparent that the rRMSE value for IOV_CL_ or IOV_V_ in one OCC was higher in sampling scheme (a) than in (b) (IOV_CL_, sampling scheme (a): 72.2%, sampling scheme (b): 27.6%). For a power outcome of > 80% the highest rRMSE value of the implemented IOV was 39.1% (IOV25_V_, sampling scheme (b), one OCC).

**Table 5:** rRMSE values of IOV when truly present for the two sampling schemes (a: 0.5, 2, 8, 22 h after dose, b: 0, 0.5, 2, 8, 22 h after dose) and two magnitudes of IOV (25%, 75%) on parameters CL, V and k_a_

Sampling	OCC	SIM with IOV_CL_		SIM with IOV_V_		SIM with IOV_ka_	
		25%	75%	25%	75%	25%	75%
		EST with IOV_CL_		EST with IOV_V_		EST with IOV_ka_	
		rRMSE [%]	rRMSE [%]	rRMSE [%]	rRMSE [%]	rRMSE [%]	rRMSE [%]
a	1	72.2	24.2	58.8	22.0	116.7	57.1
a	2	17.9	11.4	16.9	11.8	25.3	13.3
a	3	12.7	10.8	12.1	10.2	17.3	9.3
b	1	27.6	15.4	39.1	18.2	113.6	34.6
b	2	13.4	11.8	15.8	12.8	23.7	13.8
b	3	10.4	10.8	11.4	11.7	17.3	10.4

### Identification of correct IOV

The mean ΔOFV of the different simulation and estimation model combinations for IOV25 and IOV75 are shown in Tables 6 and 7, respectively. Overall, the results from IOV75 scenarios show the same tendencies as the IOV25 ones. IOV75 led to higher ΔOFV with highest values when the estimation and simulation model were the same.

For one scenario (one OCC, IOV25_ka_ (b)) none of the tested IOVs led to a significant mean ΔOFV. In the IOV25_ka_ scenario (sampling scheme (a), one OCC) only estimation with IOV_V_ led to a significant ΔOFV. When two different IOVs resulted in significant ΔOFV, the higher ΔOFV identified the correct IOV in most of the scenarios.

**Table 6:** Mean delta objective function values (dOFV) from estimation of IIV_only_ compared to estimation of IOV25_CL_/IOV25_V_/IOV25_ka_ for the two sampling schemes (a: 0.5, 2, 8, 22 h after dose, b: 0, 0.5, 2, 8, 22 h after dose) after simulation with IOV25_CL_/IOV25_V_/IOV25_ka_

Sampling	OCC	SIM with IOV25_CL_			SIM with IOV25_V_			SIM with IOV25_ka_		
		EST with IIV_only_ and			EST with IIV_only_ and			EST with IIV_only_ and		
		IOV_CL_	IOV_V_	IOV_ka_	IOV_CL_	IOV_V_	IOV_ka_	IOV_CL_	IOV_V_	IOV_ka_
		dOFV	dOFV	dOFV	dOFV	dOFV	dOFV	dOFV	dOFV	dOFV
a	1	3.13	1.09	0.20	0.60	4.48	0.57	0.24	0.48	0.19
a	2	86.60	0.07	0.01	0.01	108.59	70.64	0.03	17.90	29.56
a	3	171.49	0.04	−0.00	−0.01	205.53	131.90	0.02	34.45	57.32
b	1	34.12	4.26	2.74	3.71	11.31	2.76	1.04	0.57	0.64
b	2	136.79	0.96	0.01	1.08	118.23	71.86	0.13	16.80	30.44
b	3	252.35	0.91	−0.00	0.84	219.35	132.58	0.01	32.8	58.37

**Table 7:** Mean delta objective function values (dOFV) from estimation of IIV_only_ compared to estimation of IOV75_CL_/IOV75_V_/IOV75_ka_ for the two sampling schemes (a: 0.5, 2, 8, 22 h after dose, b: 0, 0.5, 2, 8, 22 h after dose) after simulation with IOV75_CL_/IOV75_V_/IOV75_ka_

Sampling	OCC	SIM with IOV75_CL_			SIM with IOV75_V_			SIM with IOV75_ka_		
		EST with IIV_only_ and			EST with IIV_only_ and			EST with IIV_only_ and		
		IOV_CL_	IOV_V_	IOV_ka_	IOV_CL_	IOV_V_	IOV_ka_	IOV_CL_	IOV_V_	IOV_ka_
		dOFV	dOFV	dOFV	dOFV	dOFV	dOFV	dOFV	dOFV	dOFV
a	1	43.83	13.56	2.47	1.21	45.76	6.52	0.66	2.58	3.47
a	2	517.25	1.62	0.00	−76.83	602.30	441.49	−6.63	169.84	259.80
a	3	967.21	4.58	0.00	−43.02	> 1000	860.67	−0.06	317.80	490.74
b	1	269.22	7.16	2.55	11.58	113.22	40.09	4.03	6.50	10.64
b	2	870.84	9.07	-0.05	1.94	684.36	501.66	0.06	161.53	269.76
b	3	> 1000	18.95	-0.04	1.13	> 1000	975.87	−0.02	301.28	503.90

### Impact of neglecting IOV

**Fig. 3:**
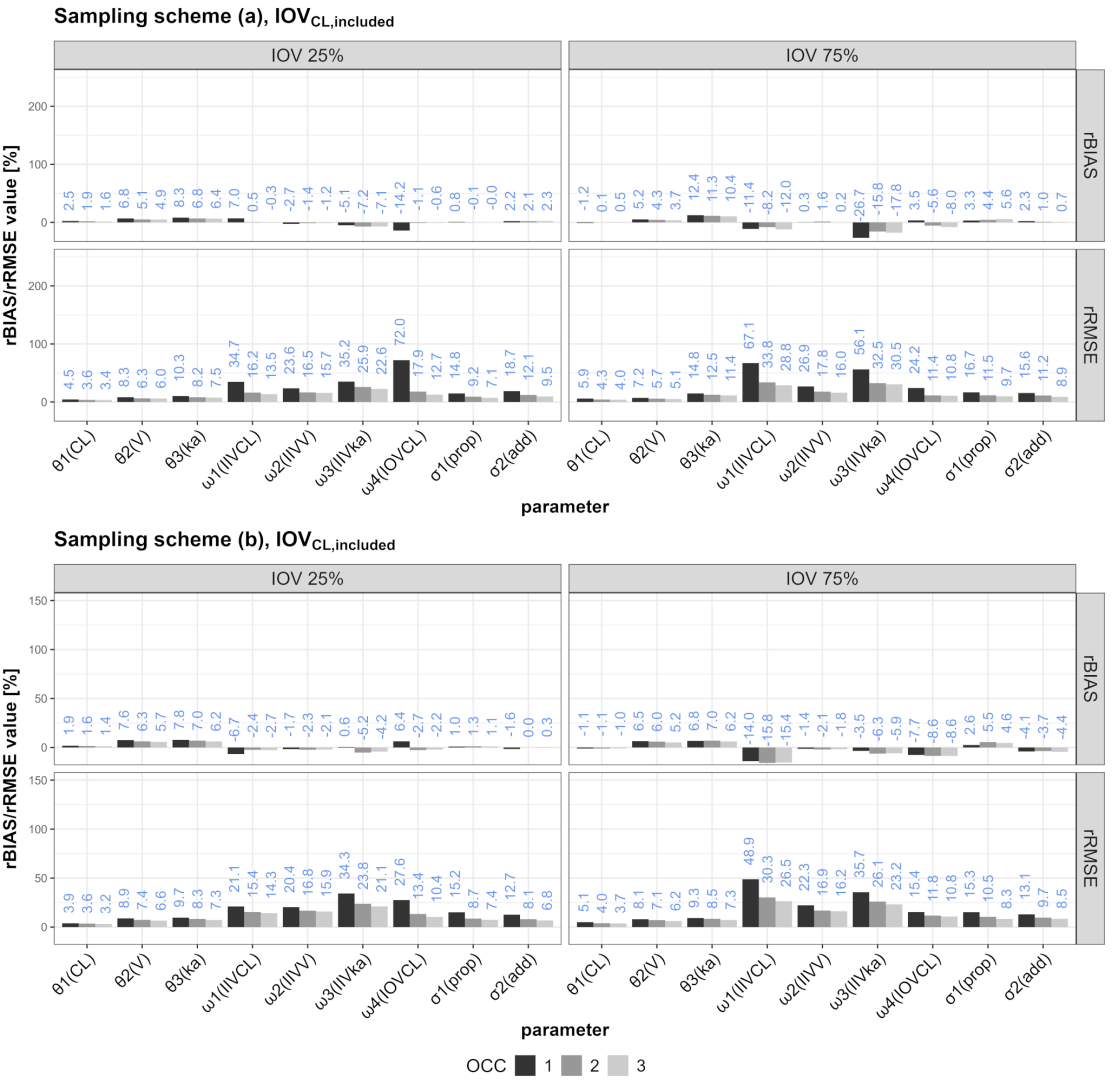
rRMSE and rBIAS values for all SSEs including one to three OCCs in which IOV on CL was included in the simulation and the estimation

**Fig. 4:**
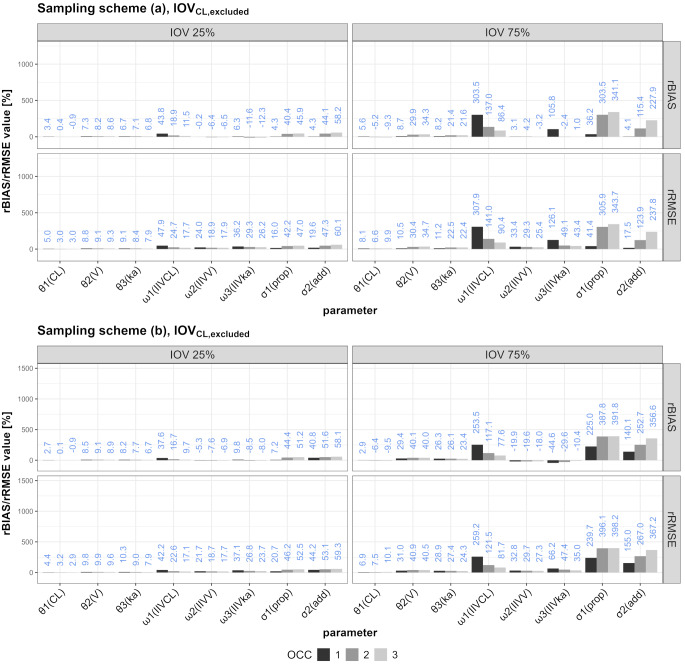
rRMSE and rBIAS values for all SSEs including one to three OCCs in which IOV on CL was included in the simulation, but neglected in the estimation

The rBIAS and rRMSE values for the different scenarios are illustrated in Figs. 3 and 4 (IOV_CL_) or the supplementary files (IOV_V_ and IOV_ka_, Figure S3-S6).

Comparing the behavior of rBIAS and rRMSE values in the scenarios in which one of three different IOVs was ignored, the following can synoptically be stated: Ignoring IOV_CL_ mostly affected the proportional error (increasing from one to three OCCs) and IIV_CL_ (decreasing from one to three OCCs). Ignoring IOV_V_ mostly affected IIV_ka_ and the proportional error, while IIV_V_ was also affected but to lesser extent. Ignoring IOV_ka_ mostly affected rBIAS and rRMSE values of IIV_ka,_ while the proportional error was also affected but to a lower degree. Generally, the sampling schemes performed similarly, but the results were more marked for IOV75 in some scenarios (e.g. IOV_CL, excluded_).

### Minimal sample size investigation

**Fig. 5:**
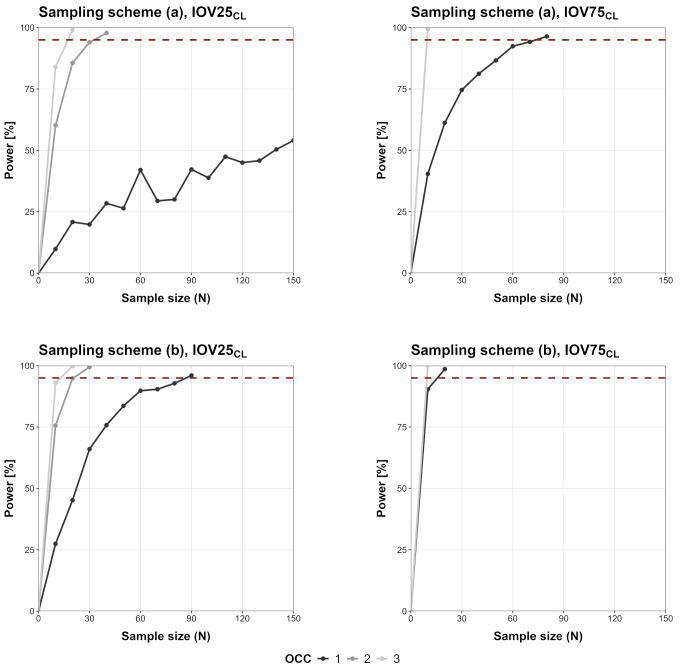
SSE-based power curves for IOV_CL_ scenarios (‘full model’ including IOV_CL_ and ‘reduced model’ not including IOV/IIV_only_), dotted line marks 95% power outcome

**Fig. 6:**
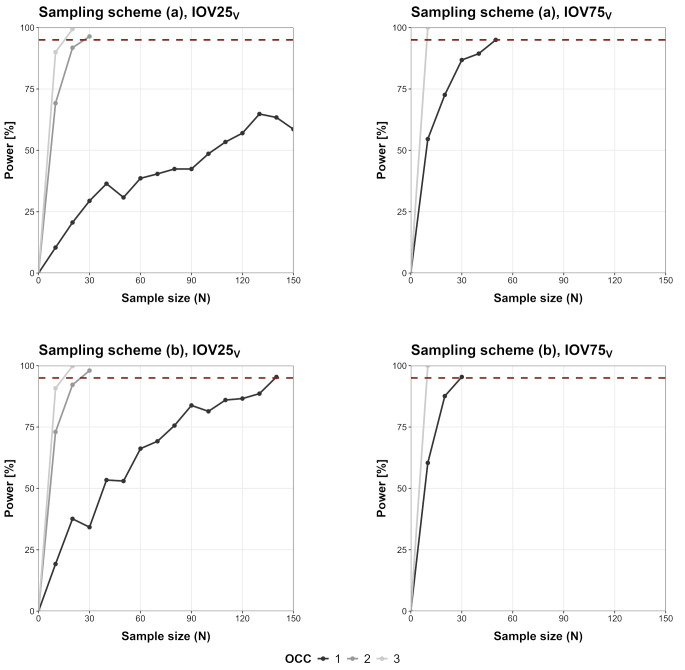
SSE-based power curves for IOV_V_ scenarios (‘full model’ including IOV_V_ and ‘reduced model’ not including IOV/IIV_only_), dotted line marks 95% power outcome

**Fig. 7:**
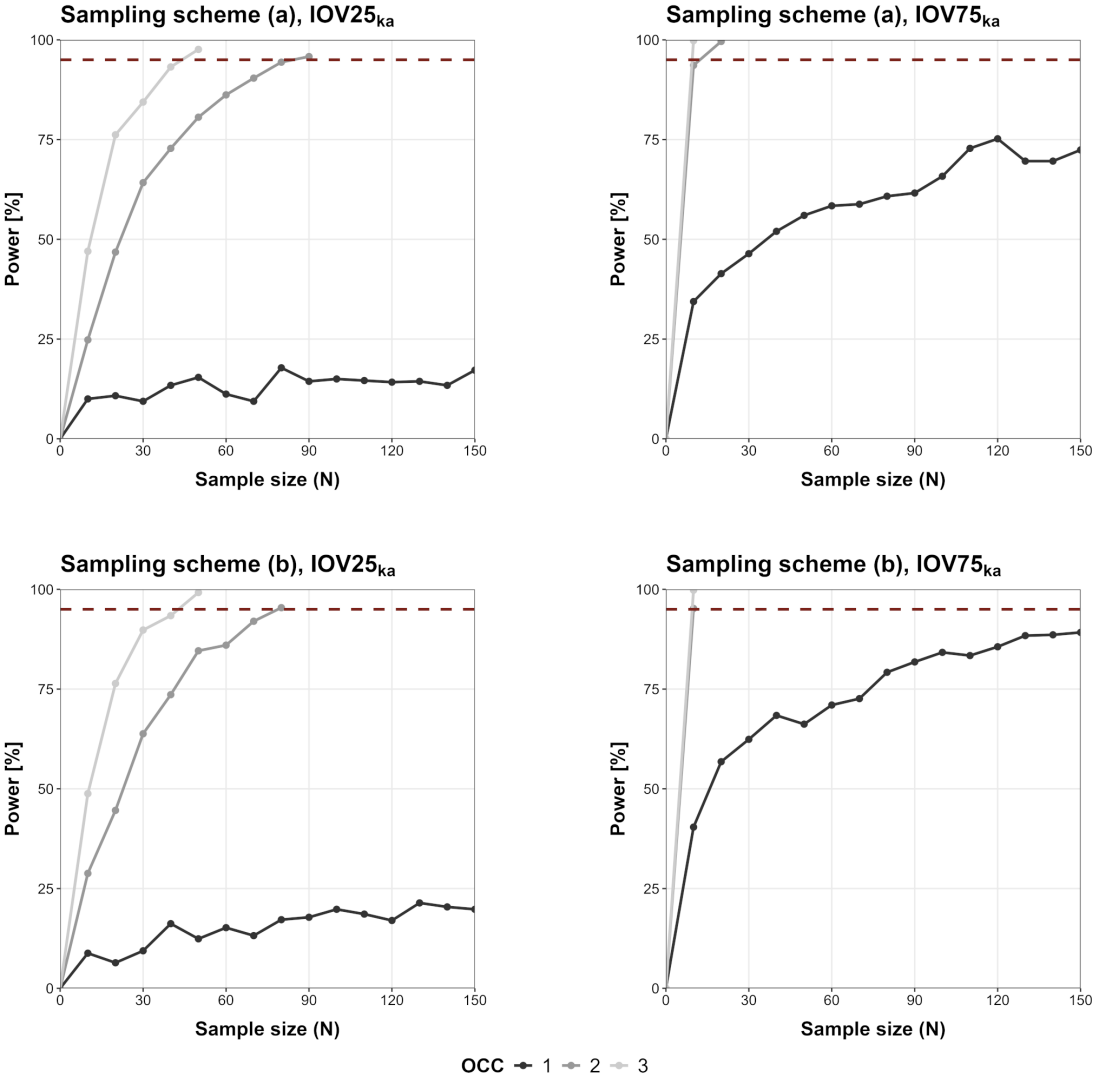
SSE-based power curves for IOV_ka_ scenarios (‘full model’ including IOV_ka_ and ‘reduced model’ not including IOV/IIV_only_), dotted line marks 95% power outcome

The SSE-based power curves in relation to the number of study subjects are shown in Fig. 5 (IOV_CL_), Fig. 6 (IOV_V_) and Fig. 7 (IOV_ka_). For IOV75_V_ and IOV75_CL_ less than 150 patients would be needed to reach 95% power even when only one OCC was observed. Overall, IOV25 led to an increased number of required patients compared to the IOV75 scenarios. In some scenarios (IOV75_CL_/IOV75_V_/IOV75_ka_, two and three OCCs, sampling (a) and (b)) only 10 patients would be required for a detection of IOV with high power (> 95%). In some scenarios sampling in one OCC was not sufficient to reach 95% power (IOV25_CL_, sampling scheme (a); IOV25_V_, sampling scheme (a); IOV25_ka_ sampling (a) and (b), IOV75_ka_, sampling (a) and (b)).

### AUC calculation

**Fig. 8:**
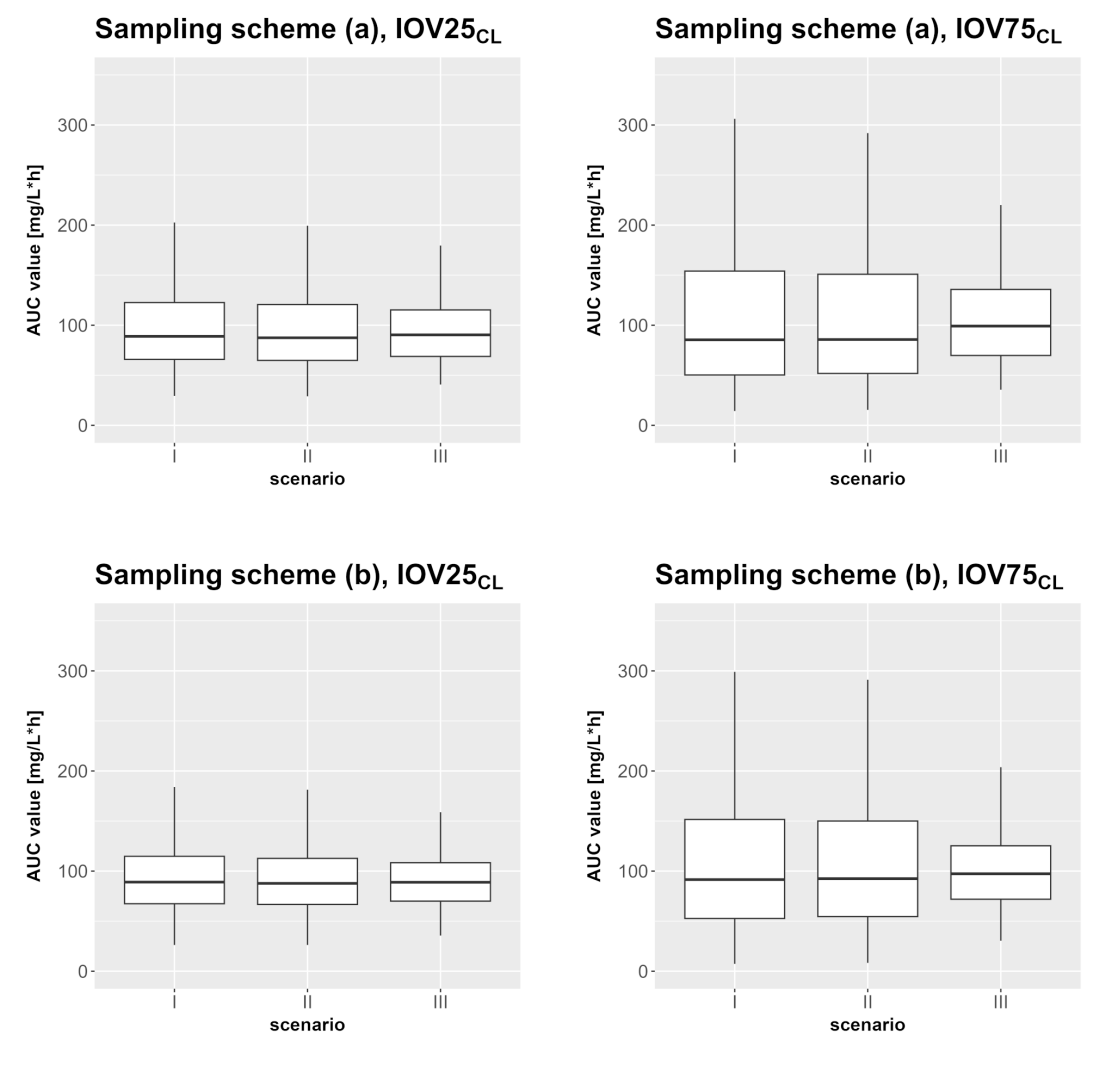
AUC values for scenarios I to III (I: true model, II: true model with final estimates from SSE, III: mis-specified IIV_only_ with final estimates from SSE) for IOV25_CL_ and IOV75_CL_ observed in three OCCs

The results of the different AUC calculations for three OCCs are shown in Fig. 8 (one OCC and two OCC in supplementary files, Figure S7 and S8). While the models that considered IOV during the estimation step (Fig. 8, II) resembled the true AUC distribution (Fig. 8, I) well, the AUC distribution was underestimated when IOV was neglected during the estimation (Fig. 8, III). For example, the 2.5^th^ to 97.5^th^ percentile for I (IOV75_CL_, sampling scheme (a)) ranges from 22.05 to 356.09 mg/L∙h. The values for II are very similar to that (23.47 to 334.44 mg/L∙h). In contrast to that, AUC values from III have a smaller range with a higher lower value and a smaller upper value compared to I or II (III: 43.17–239.66 mg/L∙h).

## Discussion

The present study investigated the impact of IOV on estimated parameters under typical optimized phase II sparse sampling designs. In particular, the impact of sampling across several dosing occasions on power, type I error, IOV model selection as well as the consequences on simulated exposure was investigated. The detection of IOV improved significantly with more OCCs, particularly noticeable from one to three OCCs Interestingly, IOV could be detected from a single OCC for CL and V in both sampling schemes if it was high (75%, power > 99.8%). IDs with very high IOV values in certain occasions play a decisive role in the high power outcome even in one observed OCC scenarios. Figure S9 illustrates the differences between IPRED and CL over time when an IOV_CL_ model (IOV25, IOV75) is used for simulation compared to an IIV_only_ model. Simulations with a model that considers IIV but no IOV result in one value for CL for the whole observed timeframe. C_min_ and C_max_ values are the same in every occasion for one individual. When a model including IOV is used for simulations the value for CL changes between the different occasions (bigger differences for higher IOV magnitude). For IOV25_CL_ the amplitude of the concentration profiles changes between occasions (more recognizable for IOV75). Hence, the differences between C_max_ and C_min_ cannot be described across all individuals by a one compartment model structure without IOV. Type I error rates were generally below 5%, indicating a low risk to falsely include an IOV when it is not present. Alpha-calibration led to critical values below 3.84 indicating that estimation of the variance of IOV was associated with less than one degree of freedom in this study. Common phase II sampling schemes provide a large enough sample size to detect an IOV if sampling is done in two OCCs (100% power), while the power for sampling in one OCC without trough sampling from the previous occasion is unreliable and only exceeds 95% for high effect sizes in some cases. It was not always possible to identify the correct IOV solely based on ΔOFV, as in some scenarios more than one IOV or no IOV had a significant ΔOFV and in one scenario an IOV resulted in a significant ΔOFV that was not included in the simulation model. However, the highest significant ΔOFV identified the correct IOV with only one exception. We were able to comprehend the influence of the ignored IOV on the estimation of the model parameters and the variabilities that were included in the model. Based on these results, sampling scheme (b) seems preferable over (a), as it generally leads to smaller rBIAS/rRMSE values in most of the evaluated scenarios. Of note, the results of the SSEs were independent of varying starting values in the estimation step. Minimal sample size investigations revealed that in all of the two and three OCC scenarios, less patients than the initial 150 patients would be needed for a detection of IOV with high power. The AUC simulations revealed that using a mis-specified model will lead to high differences in the AUC distribution (decrease in interquartile range (2.5^th^– 97.5^th^) by factor ~ 1.5). The high scattering of the AUC values when IOV is truly present and accounted for in the model cannot be reached by the mis-specified models. Neglecting an IOV that is truly present would therefore have a direct impact if AUC is used in decision making e.g. AUC-targeted dosing during clinical drug development [13].

During the methodological elaboration of our study, the Monte-Carlo Mapped Power method (MCMP) from PsN was used to determine the minimal sample size. Vong et al. [14] reported, that the MCMP method shows a lack of precision for powers < 20%. In our pretests, even with power > 40% an imprecise result was observed that was not reproducible when a different seed was used for the simulation. Therefore, we decided to evaluate the correlation of sample size to power with the conventional SSE tool of PsN.

Some of our results can be compared to the findings of Karlsson and Sheiner [1], because they also evaluated the influence of ignoring an IOV on parameter estimation. Both simulation studies share a number of key results, but we can also point out certain differences. Our study also investigated IOV on the oral absorption rate constant, while Karlsson and Sheiner focused on the slightly less complex i.v. administration [1]. IOV on oral absorption might be a highly relevant IOV in clinical practice. Instead, while we only evaluated models containing one IOV at a time, Karlsson and Sheiner also included a model with two IOVs in their study [1]. Overall, our simulation study adds the power and type I error calculations, the evaluation of the ability to detect the correct IOV as well as the power correlated sample size examination. Additionally, we evaluated the consequences of using a mis-specified model/ignored IOV on simulated AUC distributions in different scenarios as an interpretable clinical example of application.

Karlsson and Sheiner concluded that ignoring an IOV leads to positive bias on the estimates of the residual error and the IIV [1]. Similarly, we saw positive rBIAS values on the residual error and IIV, but our study adds that it depends on the scenario/IOV to which extend each part is biased. They also made statements about the relation of the size of IOV compared to IIV. For IOV being smaller than IIV, the bias in IIV_CL_ and IIV_V_ were diminutive while for higher IOV than IIV they reported a fivefold rise in biases [1]. Likewise, we detected noticeably higher rBIAS values in IOV75 scenarios in comparison to IOV25 scenarios (rBIAS of IIV_CL_ for IOV25_CL, included_/IOV75_CL, included_: −0.3%/−12.0% (three OCCs), rBIAS of IIV_V_ for IOV25_V, included_/IOV75_V, included_: −5.2%/−25.1% (one OCC)).

One reason Karlsson and Sheiner assign for the importance of modeling IOV is the importance of IOV for decision-making e.g. in regard to study design [1]. In our case, we could make a decision on which sampling scheme to choose for our fictitious study (sampling (a) or (b)). When we include an IOV in our model, we would prefer sampling scheme (b) under the permission that one additional sample is feasible in the context of the clinical study, because sampling scheme (b) resulted in more favorable power and rRMSE/rBIAS values. In case of falsely ignoring the IOV that is truly present decision-making gets more difficult. For IOV25 scenarios, in which the IOV was smaller than IIV, the differences between the sampling schemes were marginal. The IOV75 scenarios instead showed larger shifts in rBIAS/rRMSE values of certain parameters/variabilities. Regarding sample sizes, sampling scheme (b) would be preferred, as less patients would effectively be required to reach a certain power level. The advantages and therein justified necessity of three observed OCCs compared to two OCCs are debatable.

The importance of IOV has been emphasized by several publications approaching this topic from various perspectives ever since [1]. Lalonde et al. focused on PD parameters as they compared methods of evaluating population dose-response and relative potency [2]. They described the effect on parameter estimation when ignoring IOV on PD parameters [2]. Overestimation of the residual variability and biased parameter estimates were reported as a result of ignoring IOV [2]. These results are similar to those reported by Karlsson and Sheiner [1] and us. Koehne-Voss et al. analyzed, inter alia, the impact of neglecting IOV in k_a_ on parameter estimates and their findings are in accordance with our results [15]. Ignoring IOV_ka_ led to increased/positive bias on the parameter estimates of V, k_a_ and IIV_ka_ in both studies with increasing bias values for higher IOV effect sizes.

The influence of IOV in individual optimal design has been described by Kristoffersson et al. [16]. They were able to demonstrate that including IOV in the maximum *a posteriori* Fisher information matrix had an effect on the optimal design calculation and considering IOV in the design development resulted in more precise individual parameter estimates [16]. Abrantes et al. centered their study around IOV in context of therapeutic drug monitoring; more precisely, they evaluated different approaches how to incorporate IOV in a Bayesian forecasting setup [17]. Consideration of IOV was found important and more precise doses were calculated when IOV was considered [17]. Alihodzic et al. reported that an uncertain documentation of sampling and infusion timepoints influenced the estimation of IOV and IIV [18]. Uncertain documentation of sampling time was found to impair the ability to detect an IOV [18]. Solely using simulated data, this potential source of uncertainty could be obviated in our study but should definitely be considered and evaluated when working with clinical data. Denti proposes that a “pre-dose IOV” might attenuate the influence of vague information about dosing history [8]. He recommends weighing up IIV and IOV likely to be important for each parameter individually and suggests coding datasets with e.g. OCC at the outset to facilitate testing for IIV/IOV [8].

There are limitations to our study. The high computational cost of the analysis limited the number of scenarios that could be explored and evaluated. The reported statistics are associated with uncertainty which might be a reason for certain results, differences or tendencies we cannot explain otherwise. Regarding applicability, we did not use real data, as our results are solely based on simulated data. Our study setup may not consider all factors arising when dealing with real life data. In real life there may be rough indications for a possible source of IOV that should definitely be taken into consideration when modeling of IOV is aspired. Chatelut et al. stated, that e.g. IOV of drug exposure is probably higher after clinical stages of drug development [3]. Thus, our assumptions about IOV regarding its magnitude should always be put in proportion and could be questioned with respect to real data. Investigations based on real life data may be insightful to confirm our results outside of a simulation study. As we discovered some differences in comparison to the results of Karlsson and Sheiner, including more parameters (e.g. bioavailability) or more complex models in a simulation study would be of interest, to find out more about the interrelationships of certain parameters or variabilities [1]. As a consequence, an evaluation of the influence of more complex compartmental structures is prospectively conceivable. We assumed that the model parameters are constant for the observed timeframe, therefore gradual changes in the parameters are not depicted in our simulation study. Nonetheless, it is a strength of our study that a D-optimal sampling scheme was used to provide optimal timepoints to support the structural PK model. Hence, it can be anticipated that the here obtained results may also apply to more complex PK models if the sampling schemes are also optimized to support their estimation. The type of patient population on the basis of which the study design optimization is performed must always be taken into consideration as a limiting factor of the optimization as e.g. a phase II population will exhibit higher variability than a prior phase I population. Thus far, the results expand the knowledge of which parameters might be hardly affected in the case of ignoring an IOV and therefore may be considered in risk evaluations prior to or accompanying designing a clinical study, respectively.

## Conclusion

IOV can have an influence on pharmacometric modeling and decisions derived from pharmacometric research questions of all kind. Study design should support and facilitate the identification of IOV. Even small changes e.g. within a sampling scheme can have a notable impact on the accurate detection of IOV. IOV is only one component of variability of a pharmacometric model and therefore closely linked to IIV and the residual variability. If it is not adequately considered or the study design does not allow for its correct estimation, the impact of IOV will be of multifactorial character.

## Electronic supplementary material

Below is the link to the electronic supplementary material.

Supplementary Material 1

## Data Availability

No datasets were generated or analysed during the current study.
