# Supplementary material for: Interoccasion variability in population pharmacokinetic models: identifiability, influence, interdependencies and derived study design recommendations
Source: J Pharmacokinet Pharmacodyn. 2025 Apr 11;52(2):23. doi: 10.1007/s10928-025-09966-7 (PMC11992005; doi:10.1007/s10928-025-09966-7)
Supplement: Supplementary file 1 — Supplementary Material 1 [file 10928_2025_9966_MOESM1_ESM.pdf]

Supplementary Information (SI)

Additional Figures S1 – S9

Article title:

Interoccasion variability in population pharmacokinetic models: identifiability, influence, interdependencies and derived study design recommendations

Authors:

Emily Behrens[1], Sebastian G. Wicha[1]

[1]Dept. of Clinical Pharmacy, Institute of Pharmacy, University of Hamburg, Germany

Corresponding author:

Prof. Dr. Sebastian G. Wicha

sebastian.wicha@uni-hamburg.de

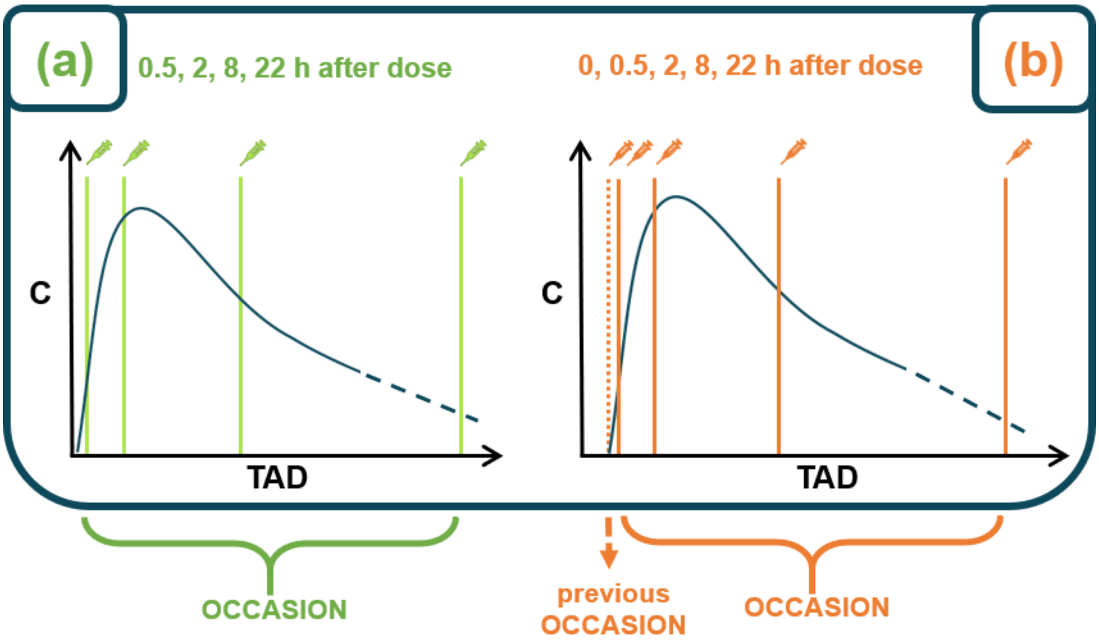

Example sampling scheme (a)

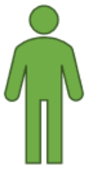

ID	TIME	EVID	AMT	DV	OCC	…
1	0	1	600	.	1	…
1	24	2	0	.	1	…
1	24	1	600	.	2	…
1	48	2	0	.	2	…
1	48	1	600	.	3	…
1	72	2	0	.	3	…
1	72	1	600	.	4	…
1	72.5	0	0	.	4	…
1	74	0	0	.	4	…
1	80	0	0	.	4	…
1	94	0	0	.	4	…
1	96	2	0	.	4	…

Example sampling scheme (b)

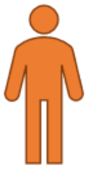

ID	TIME	EVID	AMT	DV	OCC	…
1	0	1	600	.	1	…
1	24	2	0	.	1	…
1	24	1	600	.	2	…
1	48	2	0	.	2	…
1	48	1	600	.	3	…
1	72	0	0	.	3	…
1	72.001	2	0	.	3	…
1	72.001	1	600	.	4	…
1	72.5	0	0	.	4	…
1	74	0	0	.	4	…
1	80	0	0	.	4	…
1	94	0	0	.	4	…
1	96	2	0	.	4	…

Fig. S1 Sampling schemes (a) and (b) and patient examples, T: trough

Tab. S2 Critical values after alpha-calibration

		SIM with IIV_{only}		SIM with IIV_{only}		SIM with IIV_{only}	
		25%	75%	25%	75%	25%	75%
		EST with IOV_{CL}		EST with IOV_{V}		EST with IOV_{ka}	
sampling	OCC	crit. value	crit. value	crit. value	crit. value	crit. value	crit. value
a	1	1.894	1.301	1.895	2.221	0.391	0.372
a	2	1.063	1.090	1.689	1.511	1.638	1.737
a	3	1.234	1.198	2.001	2.049	2.027	2.514
b	1	3.758	3.858	1.991	2.213	1.339	1.166
b	2	1.595	1.981	1.655	1.487	1.851	2.055
b	3	1.558	1.701	1.275	1.280	1.797	1.602

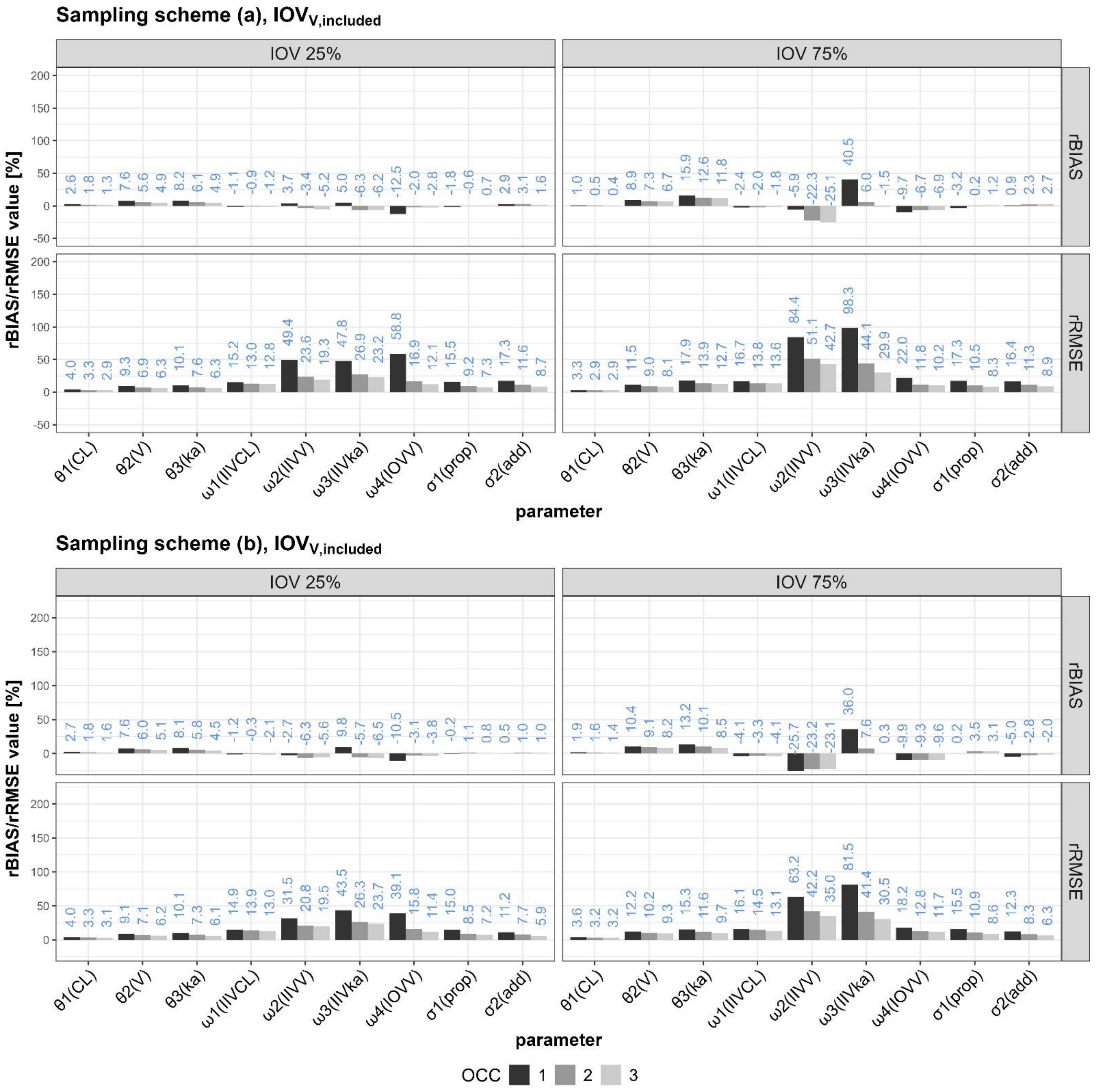

Fig. S3 rRMSE and rBIAS values for all SSEs including one to three OCCs in which IOV on V was included in the simulation and the estimation

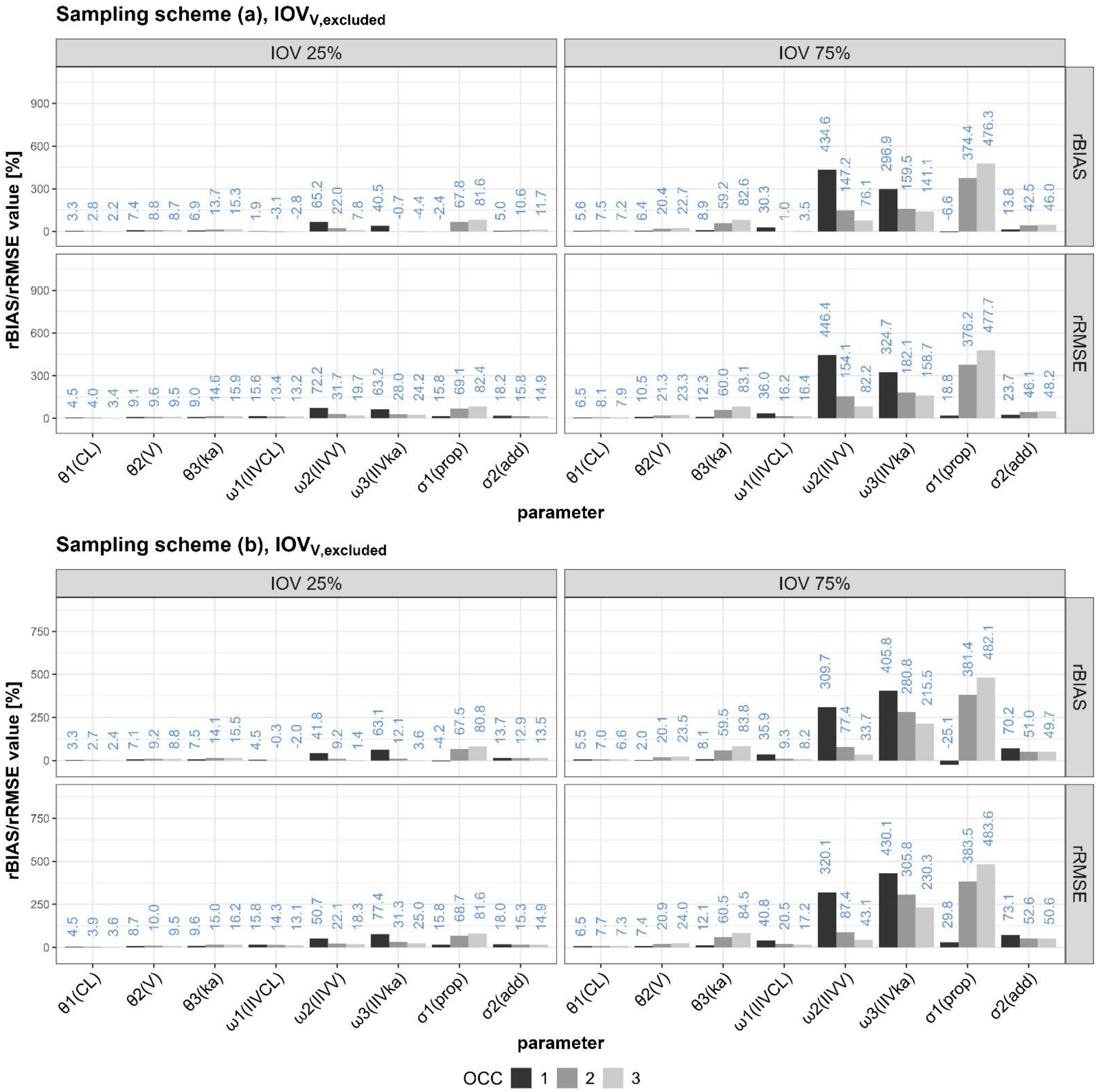

Fig. S4 rRMSE and rBIAS values for all SSEs including one to three OCCs in which IOV on V was included in the simulation, but neglected in the estimation

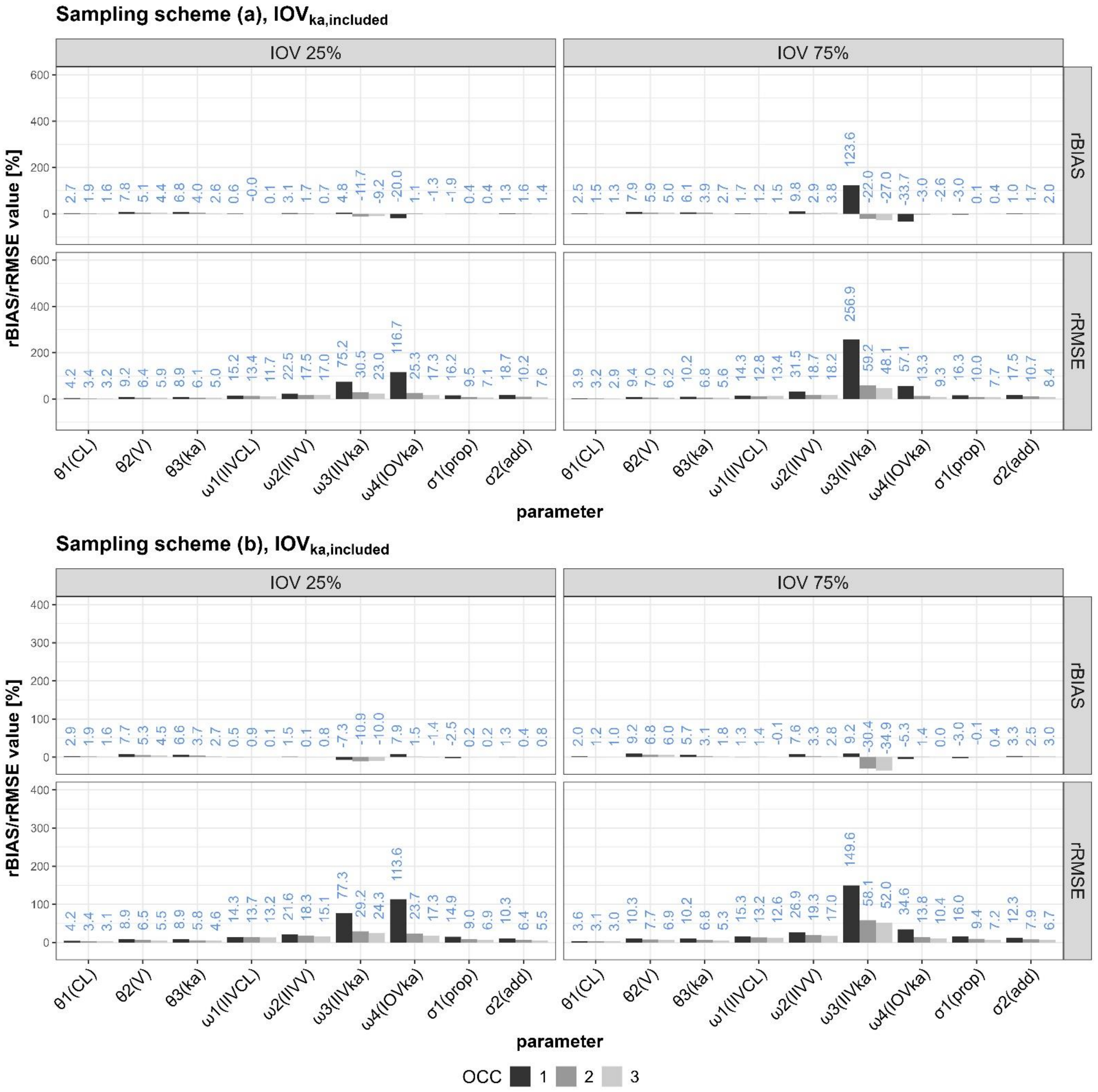

Fig. S5 rRMSE and rBIAS values for all SSEs including one to three OCCs in which IOV on k_a was included in the simulation and the estimation

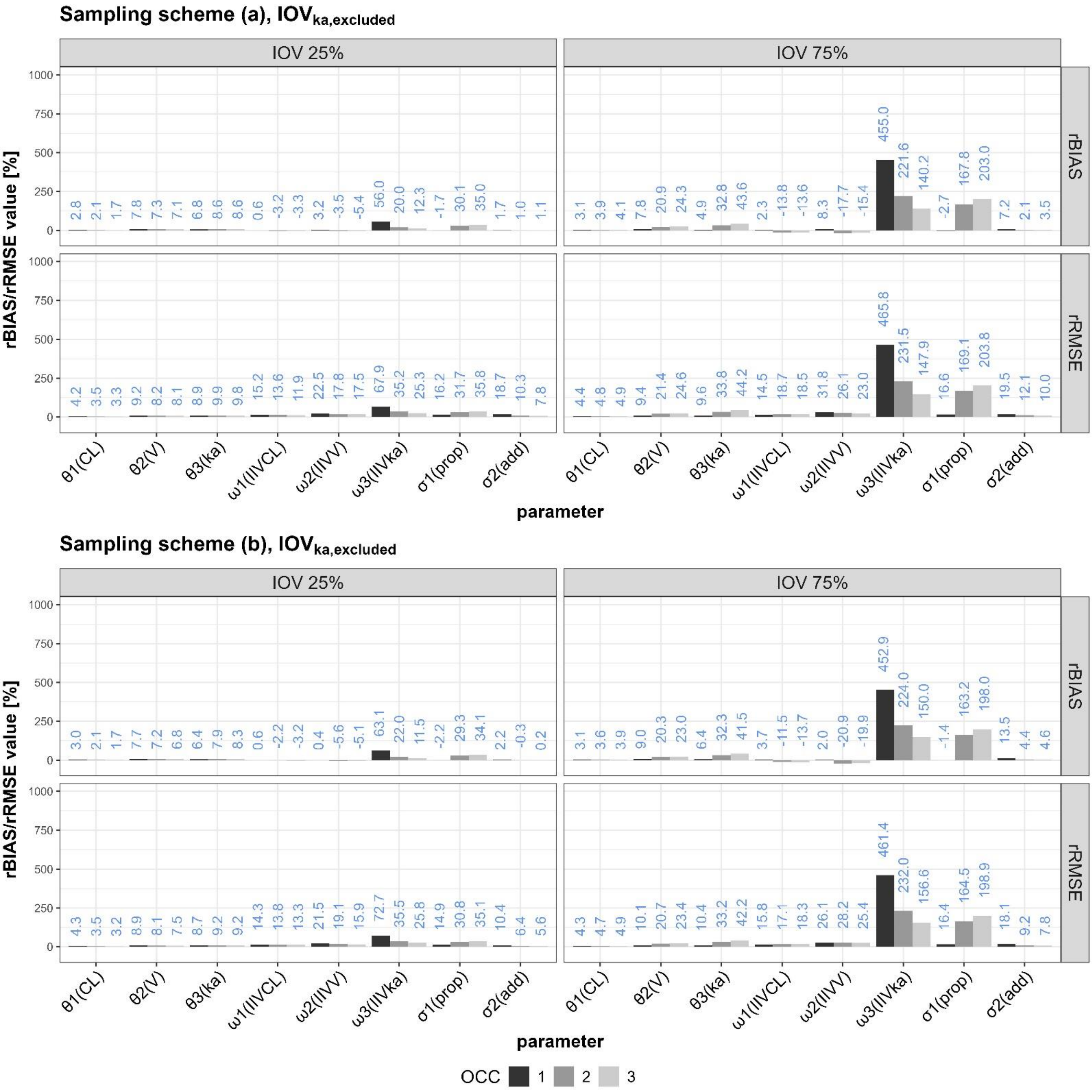

Fig. S6 rRMSE and rBIAS values for all SSEs including one to three OCCs in which IOV on k_a was included in the simulation, but neglected in the estimation

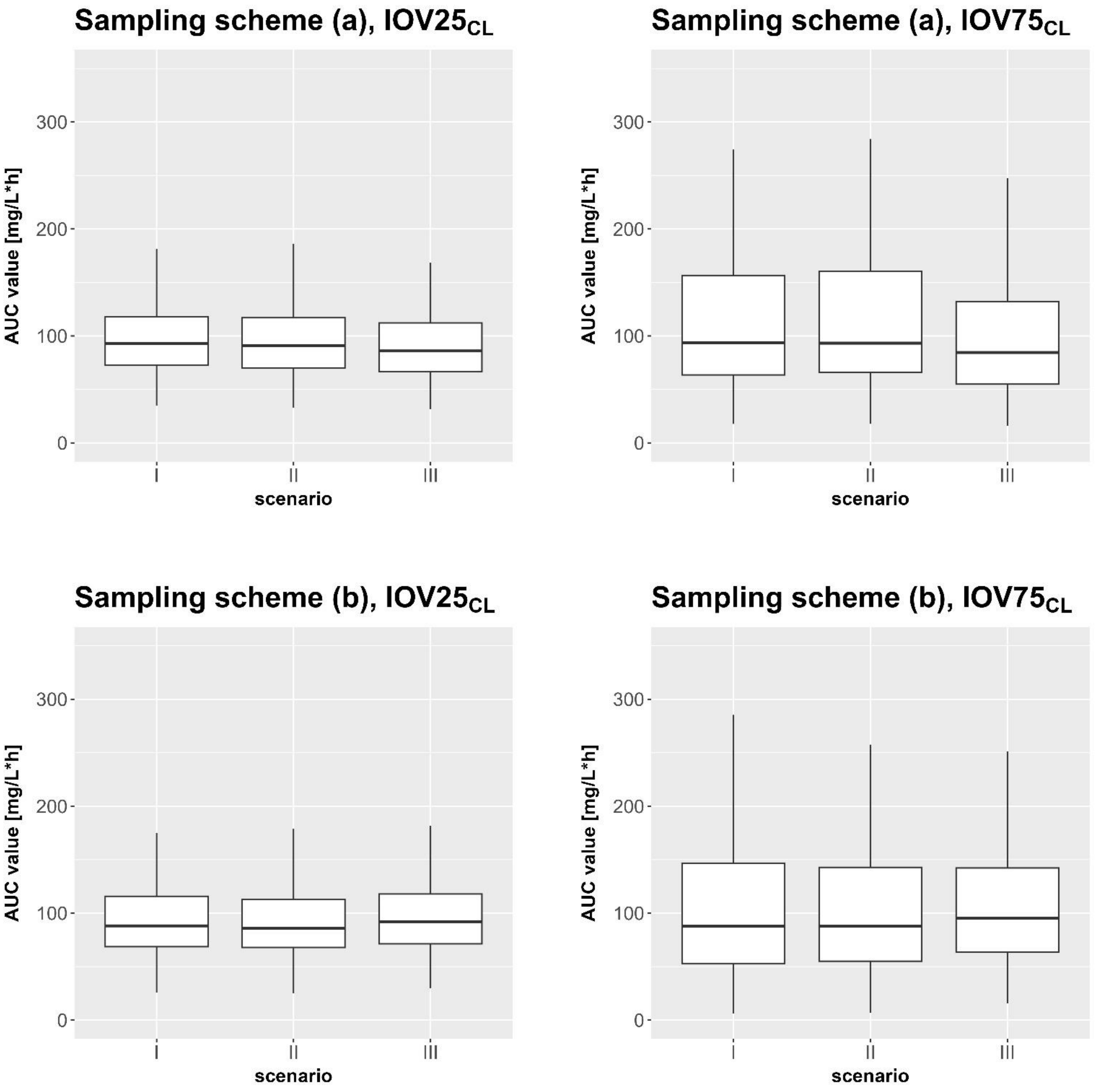

Fig. S7 AUC values for scenarios I to III (I: true model, II: true model with final estimates from SSE, III: mis-specified IIV_{only} with final estimates from SSE) for $IOV25_{CL}$ and $IOV75_{CL}$ observed in one OCC

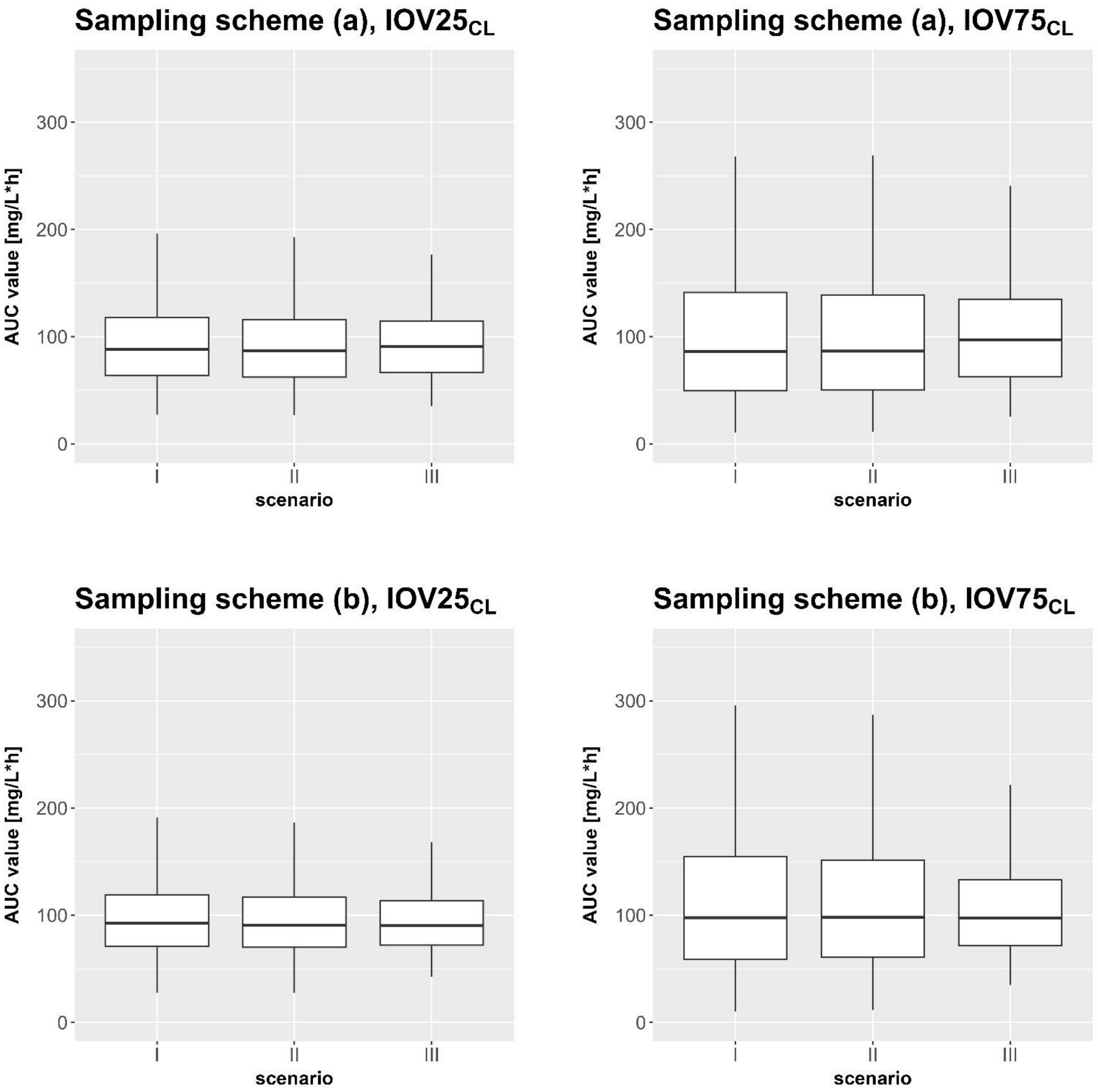

Fig. S8 AUC values for scenarios I to III (I: true model, II: true model with final estimates from SSE, III: mis-specified IIV_{only} with final estimates from SSE) for $IOV25_{CL}$ and $IOV75_{CL}$ observed in two OCCs

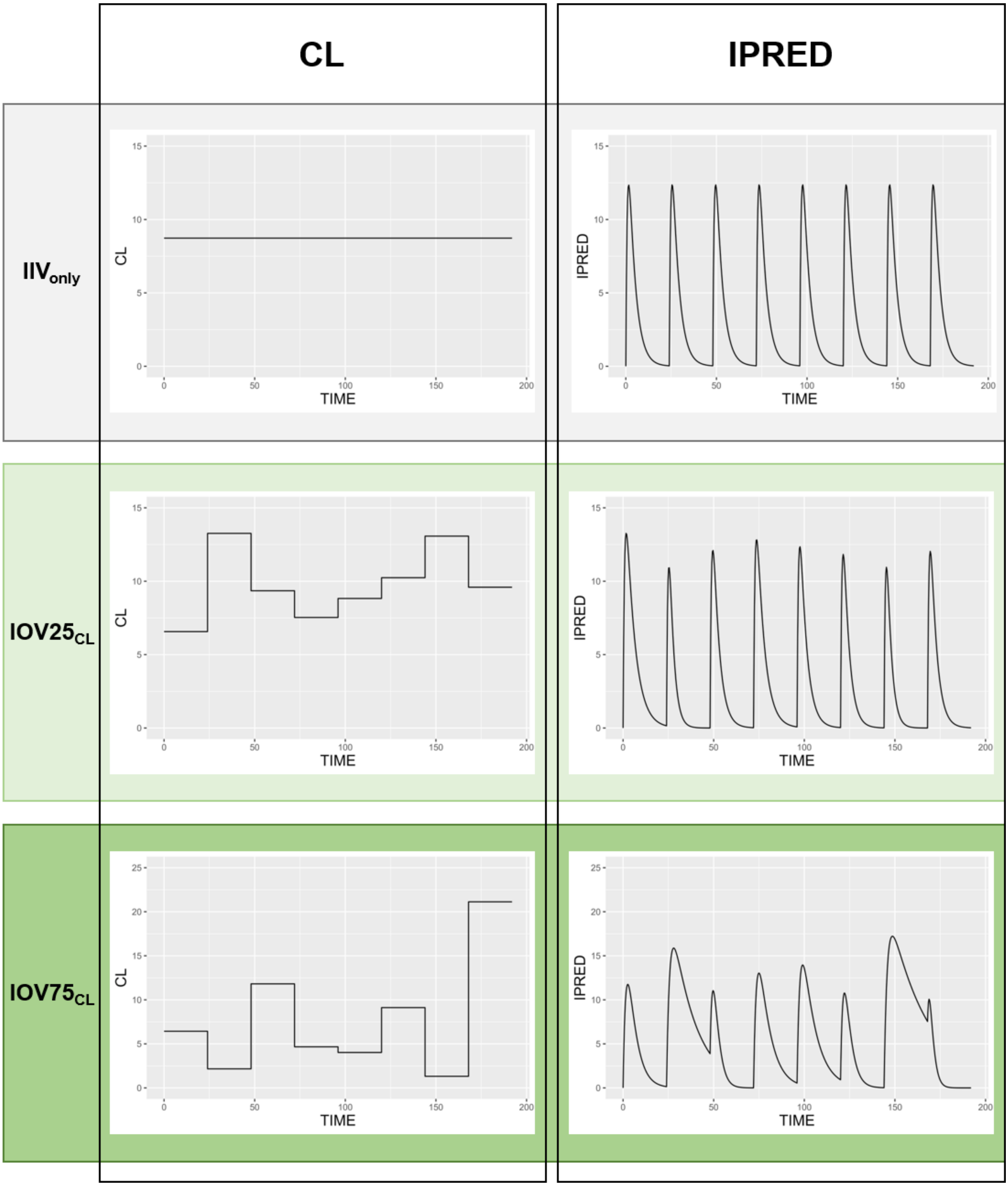

Fig. S9 IPRED vs. TIME and CL vs. TIME for simulations with a model including IIV (IIV_{only}) and models including IOV (25% IOV on CL: $IOV25_{CL}$, 75% IOV on CL: $IOV75_{CL}$) in three OCCs and sampling scheme (a)
